# 术后放疗在期Ⅰ/Ⅱ期/Ⅲ期胸腺肿瘤中的作用——ChART回顾性数据库研究结果

**DOI:** 10.3779/j.issn.1009-3419.2016.07.09

**Published:** 2016-07-20

**Authors:** 乾文 刘, 志涛 谷, 富 杨, 剑华 傅, 毅 沈, 煜程 魏, 黎杰 谭, 鹏 张, 泳涛 韩, 椿 陈, 仁泉 张, 印 李, 克能 陈, 和忠 陈, 永煜 刘, 有斌 崔, 允 王, 烈文 庞, 振涛 于, 鑫明 周, 阳春 柳, 锦 向, 媛 刘, 文涛 方, 中国胸腺肿瘤协作组成员

**Affiliations:** 1 510060 广州，中山大学附属肿瘤医院胸外科 Department of Thoracic Surgery, Guangdong Esophageal Cancer Institute, Sun Yat-sen University Cancer Center, State Key Laboratory of Oncology in South China, Collaborative Innovation Center of Cancer Medicine, Guangzhou 510060, China; 2 200030 上海，上海交通大学附属上海胸科医院 Department of Thoracic Surgery, Shanghai Chest Hospital, Shanghai Jiao Tong University, Shanghai 200030, China; 3 200080 上海，上海市第一人民医院 Department of Thoracic Surgery, Shanghai General Hospital, Shanghai Jiao Tong University, Shanghai 200080, China; 4 青岛，青岛大学医学院附属医院胸外科 Department of Thoracic Surgery, Afliated Hospital of Qingdao University, Qingdao 266001, China; 5 200032 上海，复旦大学 附属中山医院胸外科 Department of Thoracic Surgery, Zhongshan Hospital, Fudan University, Shanghai 200032, China; 6 300052 天津，天津医科大学附属总医院胸外科 Department of Endocrinology, Tianjin Medical University General Hospital, Tianjin 300052, China; 7 610041 成都，四川省肿瘤医院胸外科 Department of Thoracic Surgery, Sichuan Cancer Hospital, Chengdu 610041, China; 8 350001 福州，福建医科大学附属协和医院胸外科 Department of Thoracic Surgery, Fujian Medical University Union Hospital, Fuzhou 350001, China; 9 230022 合肥，安徽医科大学附属第一医院胸外科 Department of Thoracic Surgery, First Afliated Hospital of Anhui Medical University, Hefei 230022, China; 10 450008 郑州，郑州大学附属肿瘤医院胸外科 Department of Thoracic Surgery, Afliated Cancer Hospital of Zhengzhou University, Zhengzhou 450008, China; 11 100142 北京，北京大学附肿瘤医院胸外科 Department of Thoracic Surgery, Beijing Cancer Hospital, Beijing 100142, China; 12 200433 上海，长海医院胸心外科 Department of Cardiothoracic Surgery, Changhai Hospital, Shanghai 200433, China; 13 110042 沈阳，辽宁肿瘤医院胸外科 Department of Thoracic Surgery, Liaoning Cancer Hospital, Shenyang 110042, China; 14 130021 长春，吉林大学附属第一医院胸外科 Department of Thoracic Surgery, First Afliated Hospital of Jilin University, Changchun 130021, China; 15 610041 成都，四川大学华西医院胸外 科 Department of Thoracic Surgery, West China Hospital, Sichuan University, Chengdu 610041, China; 16 200032 上海，复旦大学附属华山医院胸外科 Department of General Surgery, Huashan Hospital, Fudan University, Shanghai 200032, China; 17 300060 天津，天津医科大学附属肿瘤医院食管癌中心 Department of Esophageal Cancer, Tianjin Cancer Hospital, Tianjin 300060, China; 18 310022 杭州，浙江省肿瘤医院胸外科 Department of Thoracic Surgery, Zhejiang Cancer Hospital, Hangzhou 310022, China; 19 330006 南昌，江西省人民医院胸外科 Department of Thoracic Surgery, Jiangxi People's Hospital, Nanchang 330006, China; 20 510060 广州，中山大学附属肿瘤医院病理科 Department of Pathology, Sun Yat-sen University Cancer Center, State Key Laboratory of Oncology in South China, Collaborative Innovation Center of Cancer Medicine, Guangzhou 510060, China

**Keywords:** 胸腺瘤, 术后放疗, 总生存, Thymic tumor, Postoperative radiotherapy, Overall surviva

## Abstract

**背景与目的:**

胸腺肿瘤术后放疗尚存在争议，此研究目的为评价术后放疗在Ⅰ期-Ⅲ期胸腺肿瘤中的作用。

**方法:**

搜索中国胸腺瘤研究协作组（Chinese Alliance of Research for Thymomas, ChART）数据库中1994年至2012年接受手术切除未行新辅助治疗的Ⅰ期-Ⅲ期胸腺肿瘤患者的资料。对临床病理资料进行单因素、多因素分析，Cox比例风险模型用于决定死亡风险比。

**结果:**

ChART数据库中Ⅰ期-Ⅲ期胸腺肿瘤共1, 546例。其中649例（41.98%）接受术后放疗。术后放疗与性别、组织学类型（World Health Organization, WHO）、胸腺切除程度、是否完全切除、Masaoka-Koga分期及辅助化疗相关。手术后辅助放疗患者5年、10年总生存和无瘤生存分别为90%和80%、81%和63%，而单纯手术者5年、10年总生存和无瘤生存分别为96%和95%、92%和90%，两组生存有统计学差异（*P*=0.001, *P*＜0.001）。单因素表明年龄、组织学分类（WHO）、Masaoka-Koga分期、是否完全切除和术后放疗与总生存相关。多因素分析提示组织学分类（WHO）（*P*=0.001）、Masaoka-Koga分期（*P*=0.029）和是否完全切除（*P*=0.003）是总生存的独立预后因素。单因素分析表明性别、重症肌无力、组织学分类、Masaoka-Koga分期、手术方式、术后放疗和是否完全切除与无瘤生存相关。多因素分析表明组织学类型（*P*＜0.001）、Masaoka-Koga分期（*P*=0.005）和是否完全切除（*P*=0.006）是无瘤生存的独立预后因素。亚组分析表明不完全切除患者接受术后放疗可以提高总生存和无瘤生存（*P*=0.010, *P*=0.017）。然而，完全切除者接受术后放疗则会降低总生存和无瘤生存（*P*＜0.001, *P*＜0.001）。

**结论:**

此回顾性研究表明不完全切除Ⅰ期-Ⅲ期胸腺肿瘤患者术后放疗可以提高总生存和无瘤生存。但是，对于完全切除患者，术后放疗总体上并未显示出生存获益。

## Abstract (en)

**Background and objective:**

Postoperative radiotherapy (PORT) for thymic tumor is still controversial. The object of the study is to evaluate the role of PORT for stage Ⅰ/Ⅱ/Ⅲ thymic tumor.

**Methods:**

The database of Chinese Alliance of Research for Thymomas (ChART) was retrieved for patients with stage Ⅰ/Ⅱ/Ⅲ thymic tumor who underwent surgical therapy without neoajuvant therapy between 1994 and 2012. Univariate and multivariate survival analyses were performed. Cox proportional hazard model was used to determine the hazard ratio for death.

**Results:**

1, 546 stage Ⅰ/Ⅱ/Ⅲ patients were identifed from ChART database. Among these patients, 649 (41.98%) underwent PORT. PORT was associated with gender, histologic type (World Health Organization, WHO), surgical extent, complete resection, Masaoka stage and adjuvant chemotherapy. The 5-yr and 10-yr overall survival (OS) rates and disease-free survival (DFS) rate for patients underwent surgery followed by PORT were 90% and 80%, 81% and 63%, comparing with 96% and 95%, 92% and 90% for patients underwent surgery alone (*P*=0.001, *P* < 0.001) respectively. In univariate analysis, age, histologic type (WHO), Masaoka stage, completeness of resection, and PORT were associated with OS. Multivariable analysis showed that histologic type (WHO)(*P*=0.001), Masaoka stage (*P*=0.029) and completeness of resection (*P*=0.003) were independently prognostic factors of OS. In univariate analysis, gender, myasthenia gravis, histologic type (WHO), Masaoka stage, surgical approach, PORT and completeness of resection were associated with DFS. Multivariable analysis showed that histologic type (WHO) (*P* < 0.001), Masaoka stage (*P*=0.005) and completeness of resection (*P*=0.006) were independently prognostic factors of DFS. Subgroup analysis showed that patients with incomplete resection underwent PORT achieved the beter OS and DFS (*P*=0.010, 0.017, respectively). However, patients with complete resection underwent PORT had the worse OS and DFS (*P* < 0.001, *P* < 0.001, respectively).

**Conclusion:**

The current retrospective study indicated that PORT afer incomplete resection could improve OS and DFS for patients with stage Ⅰ/Ⅱ/Ⅲ thymic tumor. But for those afer complete resection, PORT may not help improve prognosis on the whole.

胸腺肿瘤包括胸腺瘤和胸腺癌是前纵隔最常见的原发恶性肿瘤，比较少见，常无症状生长。根据SEER数据库，美国胸腺肿瘤每年发生率为0.13/10万^[1]^。超过30%的胸腺肿瘤伴随重症肌无力^[2]^。手术是胸腺肿瘤最重要的治疗方式。整个肿瘤的完全切除是独立预后因素^[3-6]^。国际胸腺肿瘤协作组（International Thymic Malignancy Interest Group, ITMIG）推荐整个胸腺和周围组织的整块切除以达到完全切除^[7]^。局部复发是术后的主要失败模式^[3, 5, 8, 9]^。完全切除者复发率低于非完全切除者，生存期胸腺肿瘤包括胸腺瘤和胸腺癌是前纵隔最常见的原发恶性肿瘤，比较少见，常无症状生长。根据SEER数据库，美国胸腺肿瘤每年发生率为0.13/10万^[1]^。超过30%的胸腺肿瘤伴随重症肌无力^[2]^。手术是胸腺肿瘤最重要的治疗方式。整个肿瘤的完全切除是独立预后因素^[3-6]^。国际胸腺肿瘤协作组（International Thymic Malignancy Interest Group, ITMIG）推荐整个胸腺和周围组织的整块切除以达到完全切除^[7]^。局部复发是术后的主要失败模式^[3, 5, 8, 9]^。完全切除者复发率低于非完全切除者，生存期为单纯手术就足够了。有关胸腺肿瘤的认识多来自于回顾性、单中心研究。目前尚无前瞻性随机研究评价术后放疗对胸腺恶性肿瘤的作用。中国胸腺瘤研究协作组（Chinese Alliance of Research for Thymomas, ChART）成立于2012年，旨在多中心合作促进胸腺肿瘤的治疗。已建立了来自中国18个中心的胸腺肿瘤回顾性资料数据库。本研究利用ChART数据库，探讨Ⅰ期-Ⅲ期胸腺肿瘤术后放疗的作用。

## 1 材料和方法

回顾性分析ChART数据库1994年至2012年间接受手术治疗胸腺肿瘤患者的资料。肿瘤分期、手术和放射治疗资料完整者纳入分析。排除接受新辅助治疗、合并其他恶性肿瘤和仅行肿瘤活检患者。所有患者根据Masaoka-Koga分期系统^[22]^重新分期。组织学分类参照2004年世界卫生组织（World Health Orgnizaition, WHO）发布的分类标准^[23]^。

用SPSS统计学软件19.0（SPSS Inc, Chicago, IL）进行统计分析。连续性变量用*Student’s t-test*进行分析。计数资料用四格表和*Pearson*卡方检验进行分析。用*Kaplan-Meier*计算生存率，用*Log-rank*检验比较生存率。用*Cox*比例风险模型进行多因素分析。*P*＜0.05为差异具有统计学意义。由于此研究仅使用去除标识患者身份的资料，免除知情同意书。

## 2 结果

ChART数据库中Masaoka-Koga分期Ⅰ期-Ⅲ期胸腺肿瘤共2, 159例。其中1, 546例有完整的分期、放射治疗和手术资料，最终被纳入研究。Masaoka-Koga分期Ⅰ期、Ⅱ期和Ⅲ期者分别为717例、318例和511例。患者的基线特征见表 1。649例（41.98%）患者接受术后放疗，897例（58.02%）接受单纯手术。两组间性别、WHO组织学分类、肿瘤大小、胸腺切除程度、是否完全切除、Masaoka-Koga分期和辅助化疗有显著差异。

**1:** 患者的基线特征 Patients' baseline characteristics

Characteristic	Surgery alone (*n*, %)*N*=897	PORT(*n*, %) *N*=649	*P* value^a^
Gender			＜0.001
Male	425(52.3)	387(47.7)	
Female	472(64.3)	262(35.7)	
Age	51.69	50.53	0.0705
Myasthenia gravis			0.161
Yes	231(61.1)	147(38.9)	
No	666(57.0)	502(43.0)	
Histologic type (WHO)			＜0.001
A	83(83.0)	17(17.0)	
AB	318(78.9)	85(21.1)	
B1	159(72.9)	59(27.1)	
B2	135(55.8)	107(44.2)	
B3	114 (40.1)	170 (59.9)	
C	74(28.0)	190(72.0)	
Carcinoid	14(40.0)	21(60.0)	
Histologic type (WHO) three classifications			＜0.001
A+AB	401(79.7)	102(20.3)	
B1+B2+B3	408(54.8)	336(45.2)	
C+NETT	88(29.4)	211(70.6)	
Surgical extent			＜0.001
Partial	182(47.6)	200(52.4)	
Total	714(61.5)	447(38.5)	
Completeness of resection			＜0.001
R0	854(61.1)	543(38.9)	
R1	27(43.5)	35(56.5)	
R2	16(18.4)	71(81.6)	
Tumor size(cm)	6.58	7.04	0.008
Masaoka stage			＜0.001
Ⅰ	535(72.6)	182(27.4)	
Ⅱ	190(59.7)	128(40.3)	
Ⅲ	172(33.7)	339(66.3)	
Adjuvant chemotherapy			＜0.001
No	854(63.9)	482(36.1)	
Yes	32(18.2)	144(81.8)	
^a^, *χ* test; WHO: World Health Organization; NETT: neuroendocrine thymic tumor. 注：本表得到版权所有者©2011-2016 Journal of Thoracic Disease复制许可。			

术后放疗5年和10年总生存及无瘤生存分别为90%和80%、81%和63%。而单纯手术5年和10年总生存及无瘤生存分别为96%和95%、92%和90%，两组生存有统计学差异（*P*=0.001, *P*＜0.001）（图 1、图 2）。单因素分析表明W HO组织学分类、Masaoka-Koga分期、是否完全切除和术后放疗与总生存相关（表 2）。多因素分析显示WHO组织学分类（*P*=0.001）、Masaoka-Koga分期（*P*=0.029）和是否完全切除（*P*=0.003）是总生存的独立预后因素，而术后放疗不是独立预后因素（表 3）。

**1:**
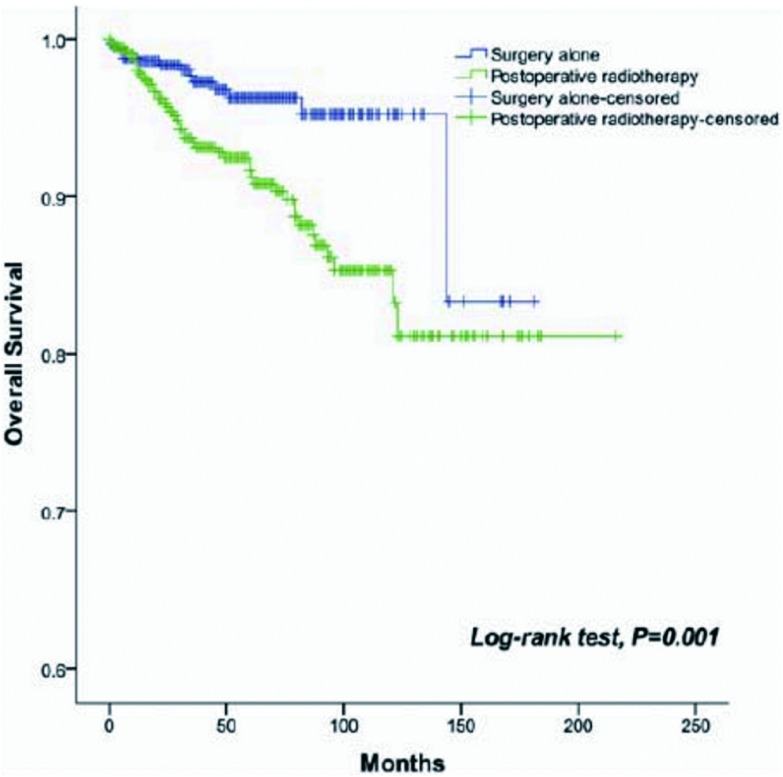
单纯手术和术后放疗患者总生存曲线。术后放疗降低了Ⅰ期-Ⅲ期胸腺上皮肿瘤患者的总生存（*P*=0.001）。 *Kaplan-Meier* overall survival curve of patients treated with surgery alone, and those treated with PORT. PORT decreased OS of stage Ⅰ/Ⅱ/Ⅲ thymic epithelial tumor (*P*=0.001).

**2:**
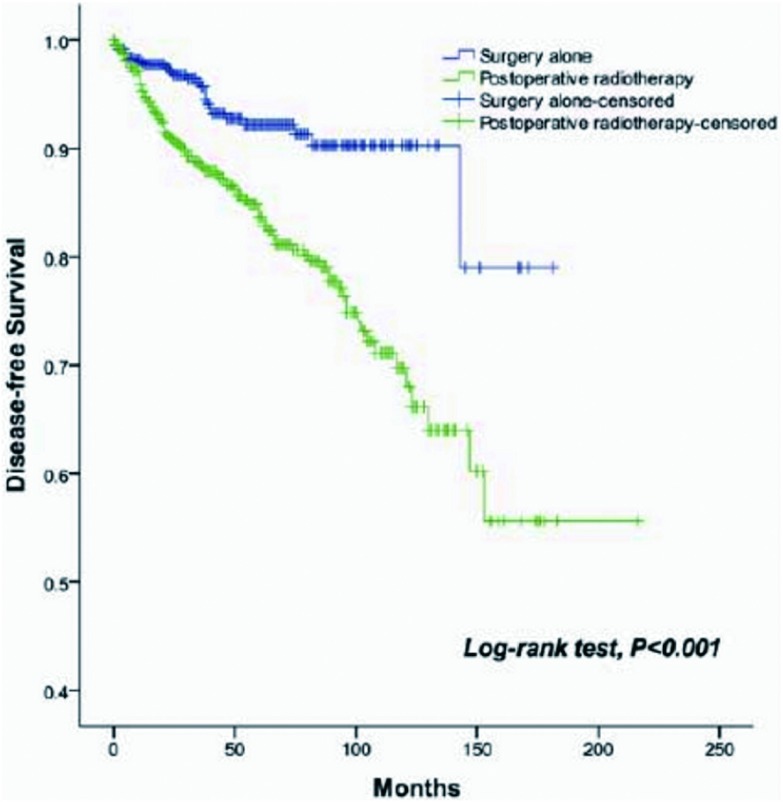
单纯手术和术后放疗患者无瘤生存曲线。术后放疗降低了Ⅰ期-Ⅲ期胸腺上皮肿瘤患者的无瘤生存（*P*＜0.001） *Kaplan-Meier* disease-free survival curve of patients treated with surgery alone, and those treated with PORT. PORT decreased DFS of stage Ⅰ/Ⅱ/Ⅲ thymic epithelial tumor (*P* < 0.001).

**2:** 总体生存的单因素分析 Univariate analysis of factors affecting overall survival

Characteristics	*P* value
Gender(Male/Female)	0.072
Age(≥50 yr/＜50 yr)	0.050
Myasthenia gravis (Yes/No)	0.081
Tumor size(≤5 cm/>5 cm)	0.524
Histologic type(WHO) (A or AB/B1 or B2 or B3/C)	＜0.001
Masaoka stage(Ⅰ/Ⅱ/Ⅲ)	＜0.001
Surgical approach(VATS/Open)	0.107
Surgical extent(Partial/Total)	0.159
PORT(No/Yes)	0.001
Completeness of resection(R0/R1+R2)	＜0.001
PORT: Postoperative radiotherapy. 注：本表得到版权所有者©2011-2016 Journal of Thoracic Disease复制许可。	

**3:** 总体生存的多因素分析 Multivariate analysis of factors affecting overall survival

Characteristics	*P* value	OR
Gender(Male/Female)	0.994	1.002
Age(＜50yr/≥50yr)	0.165	1.518
Myasthenia gravis(No/Yes)	0.811	1.117
Histologic type(WHO)(A or AB/B1 or B2 or B3/C)	0.001	
B1+B2+B3	0.073	3.226
C	0.001	8.631
Masaoka stage(Ⅰ/Ⅱ/Ⅲ)	0.029	
Ⅱ	0.124	2.425
Ⅲ	0.008	3.901
PORT	0.338	0.726
Completeness of resection(R0/R1+R2)	0.003	0.381
注：本表得到版权所有者©2011-2016 Journal of Thoracic Disease复制许可。		

单因素分析表明性别、重症肌无力、W HO组织学分类、Masaoka-Koga分期、手术方式、术后放疗和是否完全切除与无瘤生存相关（表 4）。多因素分析表明WHO组织学分类（*P*＜0.001）、Masaoka-Koga分期（*P*=0.005）和是否完全切除（*P*=0.006）是无瘤生存独立预后因素（表 5）。亚组分析表明不完全切除患者接受术后放疗较单纯手术可以提高总生存和无瘤生存（*P*值分别为0.010和0.017）。然而，完全切除者接受术后放疗则会降低总生存和无瘤生存（*P*值分别为＜0.001和＜0.001）。除了Ⅱ期患者接受放疗后无瘤生存降低外，大部分不同期别和组织学分类的患者是否接受术后放疗生存无统计学差异（表 6、表 7）。

**4:** 无瘤生存期的多因素分析 Univariate analysis of factors affecting disease-free survival

Characteristics	*P* value
Gender(Male/Female)	0.008
Age(＜50 yr/≥50 yr)	0.254
Myasthenia gravis (Yes/No)	0.002
Tumor size(≤5 cm/>5 cm)	0.094
Histologic type(WHO) (A or AB/B1 or B2 or B3/C)	＜0.001
Masaoka stage(Ⅰ/Ⅱ/Ⅲ)	＜0.001
Surgical approach(VATS/Open)	＜0.001
Surgical extent(Partial/Total)	0.629
PORT(No/Yes)	＜0.001
Completeness of resection(R0/R1+R2)	＜0.001
注：本表得到版权所有者©2011-2016 Journal of Thoracic Disease复制许可。	

**5:** 无瘤生存的多因素分析 Multivariate analysis of factors affecting disease-free survival

Characteristics	*P* value	OR
Gender(Male/Female)	0.675	0.914
Myasthenia gravis(No/Yes)	0.099	0.517
Histologic type(WHO)(A or AB/B1 or B2 or B3/C)	＜0.001	
B1+B2+B3	0.001	4.909
C	＜0.001	10.194
Masaoka stage(Ⅰ/Ⅱ/Ⅲ)	0.005	
Ⅱ	0.014	2.549
Ⅲ	0.001	3.056
Surgical approach(VATS/Open)	0.447	1.601
PORT(No/Yes)	0.971	0.991
Completeness of resection(R0/R1+R2)	0.006	0.513
注：本表得到版权所有者©2011-2016 Journal of Thoracic Disease复制许可。		

**6:** 术后放疗对无瘤生存期影响的分层分析 Stratified disease-free survival analysis of the role of PORT

Characteristics	Patients(*n*, %)	DFS		*P* value
		5-year	10-year	
R0	1, 027			＜0.001
PORT	457 (44.38)	0.86	0.70	
Surgery alone	570(55.50)	0.96	0.95	
R1+R2	99			0.017
PORT	78(78.79)	0.60	0.39	
Surgery alone	21(21.21)	0.35	0.35	
A+AB	365			0.646
PORT	89(24.38)	0.99	0.90	
Surgery alone	276(75.62)	0.98	0.98	
B1+B2+B3	549			0.053
PORT	285(51.91)	0.89	0.66	
Surgery alone	264(48.09)	0.93	0.90	
C+NETT	212			0.702
PORT	161(75.94)	0.61	0.39	
Surgery alone	51(24.06)	0.67	0.67	
Stage Ⅰ	513			0.096
PORT	155(30.21)	0.97	0.81	
Surgery alone	358(69.79)	0.98	0.97	
Stage Ⅱ	243			0.003
PORT	108(44.44)	0.85	0.66	
Surgery alone	135(55.56)	0.99	0.99	
Stage Ⅲ	370			0.728
PORT	272(73.51)	0.71	0.51	
Surgery alone	98(26.49)	0.70	0.70	
DFS: Disease free survival. 注：本表得到版权所有者©2011-2016 Journal of Thoracic Disease复制许可。				

**7:** 术后放疗总生存的分层分析 Stratified overall survival analysis of the role of PORT

Characteristics	Patients(*n*, %)	OS		*P* value
		5-year	10-year	
R0	1, 023			＜0.001
PORT	454(44.38)	0.93	0.87	
Surgery alone	569(55.62)	0.98	0.98	
R1+R2	96			0.010
PORT	77(80.21)	0.75	0.51	
Surgery alone	19(19.79)	0.59	0.30	
A+AB	365			0.285
PORT	89(24.38)	0.99	0.90	
Surgery alone	276(75.62)	1.00	1.00	
B1+B2+B3	547			0.280
PORT	285(52.10)	0.92	0.91	
Surgery alone	262(47.90)	0.95	0.95	
C+NETT	207			0.930
PORT	157(75.85)	0.80	0.53	
Surgery alone	50(24.15)	0.85	0.76	
Stage Ⅰ	511			0.067
PORT	153(29.94)	0.97	0.91	
Surgery alone	358(70.06)	0.99	0.99	
Stage Ⅱ	243			0.537
PORT	108(44.44)	0.94	0.89	
Surgery alone	135(55.56)	0.98	0.98	
Stage Ⅲ	365			0.717
PORT	270(73.97)	0.84	0.69	
Surgery alone	95(26.03)	0.85	0.79	
OS: Overall survival. 注：本表得到版权所有者©2011-2016 Journal of Thoracic Disease复制许可。				

## 3 讨论

术后放疗在胸腺肿瘤中的作用尚存在争议。局部复发是胸腺肿瘤术后最常见的失败模式。有文献报道术后放疗可以使复发率从30%降至5%^[24, 25]^。鉴于胸腺肿瘤罕见、无痛性生长的自然病程以及大部分患者死于不相关的因素，目前尚无前瞻性研究探讨术后放疗的真正作用。

本多中心研究中，从ChART数据库中筛选出1, 546例Masaoka-Koga分期Ⅰ期-Ⅲ期患者。令人遗憾的是术后放疗并未提高总生存。手术后接受放疗患者5年和10年总生存分别为90%和80%，而单纯手术者5年和10年总生存分别为96%和95%（*P*=0.001）。这可能归因于术后放疗组有较多胸腺癌、Ⅲ期以及姑息切除患者。然而，对于姑息切除患者术后放疗可以提高总生存。

现有数据表明Masaoka-Koga分期、是否完全切除以及组织学分类是独立预后因素^[3, 4, 8]^。与Detterbeck等^[3]^的*meta*分析结果一致，胸腺瘤各亚类间生存无统计学差异。本研究也表明胸腺瘤完全切除就可以获得满意的疗效，可以避免纵隔放疗带来的副反应，比如放射性肺炎、慢性肺纤维化、限制性心肌病和心包积液^[26-30]^。Mangi等^[21]^的回顾性研究表明大部分Ⅲ期患者可以获得完全切除，术后加上放疗并不降低复发率。Ⅲ期胸腺瘤患者完全切除术后辅助放疗需要慎重考虑。Utsumi等^[19]^认为A型、AB型、B1型和Masaoka-Koga分期Ⅰ期和Ⅱ期患者完全切除就足够了。另外，Ⅲ期/Ⅳ期和WHO分类为B2/B3患者术后是否放疗生存无统计学差异。Kondo等^[5]^分析了1, 320例Ⅱ期和Ⅲ期胸腺瘤，发现术后放疗并未显著降低局部复发率，也未提高侵袭性胸腺瘤的预后。完全切除是胸腺上皮肿瘤治疗中最重要的因素。Korst等^[20]^的*meta*分析表明Ⅱ期和Ⅲ期胸腺上皮肿瘤完全切除术后辅助放疗没有显著降低复发。Weksler等^[18]^利用SEER数据库报道了一项回顾性研究。这项大样本研究表明术后放疗可以提高疾病特异性生存。但是多因素分析表明术后放疗与总生存不相关。基于本研究和现有的文献资料可以看出今后关于胸腺瘤术后放疗的研究应着重于局部复发高危患者。

胸腺癌是胸腺肿瘤中最具侵袭性的类型。Ⅲ手术是可切除胸腺癌有望获得根治的治疗方式。完全切除的胸腺癌患者生存优于未接受手术治疗者。由于胸腺癌少见，缺乏高级别证据，术后化疗和放疗的作用尚不明确。Weksler等^[31]^通过SEER数据库分析了290例胸腺癌患者资料。发现术后放疗不能提高完全切除患者的总生存。完全切除肿瘤是胸腺癌的重要治疗方式。本研究中我们也发现术后放疗并不能提高胸腺癌的预后。然而，Hsu等^[32]^认为术后放疗似乎可以提高胸腺癌的预后，尽管生存差异未达到统计学意义。Omasa等^[33]^也发现术后放疗未提高Ⅱ期和Ⅲ期胸腺瘤的无复发生存和总生存，但是提高了而Ⅱ期和期胸腺癌的无复发生存。Ahmad等^[34]^发现放疗可以提高胸腺癌患者的总生存和无复发生存。

此研究表明根治性切除、WHO组织学分类和Masaoka-Koga分期是胸腺恶性肿瘤的独立预后因素。我们的研究表明术后放疗无法给完全切除的Ⅰ期-Ⅲ期胸腺肿瘤患者带来生存获益。术后放疗应应用于姑息切除的患者，可以提高其预后。由于此研究为回顾性研究，放射野和放射剂量千差万别，为明确术后放疗的真正作用，今后需针对复发高危患者进行前瞻性随机临床试验。
